# The contribution of microbial biotechnology to sustainable development goals

**DOI:** 10.1111/1751-7915.12818

**Published:** 2017-08-25

**Authors:** Kenneth Timmis, Willem M. de Vos, Juan Luis Ramos, Siegfried E. Vlaeminck, Auxiliadora Prieto, Antoine Danchin, Willy Verstraete, Victor de Lorenzo, Sang Yup Lee, Harald Brüssow, James Kenneth Timmis, Brajesh K. Singh

**Affiliations:** ^1^ Institute of Microbiology Technical University of Braunschweig Braunschweig Germany; ^2^ Laboratory of Microbiology Wageningen University Wageningen The Netherlands; ^3^ Department of Bacteriology and Immunology University of Helsinki Helsinki Finland; ^4^ Estacion Experimental del Zaidin Granada Spain; ^5^ Department of Bioscience Engineering University of Antwerp Antwerp Belgium; ^6^ Polymer Biotechnology Lab Centro de Investigaciones Biologicas Madrid Spain; ^7^ ICAN CHU Pitié‐Salpêtrière Paris France; ^8^ Center for Microbial Ecology and Technology (CMET) Ghent University Ghent Belgium; ^9^ Centro Nacional de Biotecnología Madrid Spain; ^10^ Department of Chemical and Biomolecular Engineering Korea Advanced Institute of Science and Technology (KAIST) Daejeon Korea; ^11^ Nestlé Research Centre Nutrition and Health Research Lausanne Switzerland; ^12^ MSc Health Policy Student Department of Surgery and Cancer Imperial College London UK; ^13^ Hawkesbury Institute for the Environment Western Sydney University Penrith South NSW Australia

## Abstract

The signature and almost unique characteristic of microbial technology is the exceptional diversity of applications it can address, and the exceptional range of human activities and needs to which it is and can be applied. Precisely because sustainability goals have very diverse and complex components and requirements, microbial technology has the ability to contribute substantively on many levels in many arenas to global efforts to achieve sustainability. Indeed, microbial technology could be viewed as a unifying element in our progress towards sustainability.

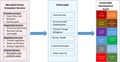

## Sustainable development involves diverse and complex approaches

Human stewardship of the planet, in particular its biosphere, is wanting: the trajectories of deterioration of critical features of the biosphere (loss of biodiversity, climate change, desertification, unbalanced N and P cycles, water quality and quantity) and the quality of the human condition (hunger, poverty, regional conflicts, refugees, human trafficking, rising health costs, diminishing urban security), on one hand, and the institution of effective corrective/mitigating actions, on the other, are divergent, so planet Earth and human life are becoming increasingly unsustainable (‘Sustainable development is development that meets the needs of the present without compromising the ability of future generations to meet their own needs’; Brundtland *et al*., 1987). To counteract this divergence, and to reorient evolution of the biosphere towards a more sustainable trajectory, internationally accepted Sustainable Development Goals (SDGs; United Nations, 2015) have been formulated by the United Nations that take into account the fact that all key biosphere and relevant human behavioural processes are interconnected, interdependent and hence must be steered via a systems approach. As a consequence, sustainable development goals are exceptionally diverse, ranging from poverty elimination to moderation of climate change, via safe cities, sustainable use of aquatic and terrestrial systems, to adoption of renewables. As all goals encompass environmental, economic and social aspects, efforts to achieve sustainability are of necessity highly complex.

## Microbes are (also) biological stewards of planetary health and sustainability

Microbes are the predominant form of life on the planet, both in numbers and total biomass. As the first forms of life on Earth, they have evolved and exhibit a spectrum of evolutionary, functional and metabolic diversity that vastly exceeds that of all other organisms in the tree of life. The ability of some microbes to inhabit hostile environments incompatible with most forms of life means that their habitats define the extent of the biosphere and delineate the barrier between the biosphere and geosphere. And, as microbes survive extreme environmental challenges, it is plausible that they allowed life, and hence the biosphere, to recover after the major catastrophes suffered by planet Earth that caused the mass extinctions (e.g. deep‐sea microbes were probably largely protected from devastating heat or cold waves in the atmosphere and could survive for very long periods in the absence of light). Microbes are very much our past and our future.

Their ubiquity throughout the biosphere and the diversity of their activities make microbes pivotal agents of planetary and ecosystem functioning: they mediate and regulate biogeochemical cycles and recycling of biological materials and waste, constitute key producers and sinks of greenhouse gases and are thus important determinants of climate change, play essential roles in soil structure and fertility, and in the quality and productivity of land, seas, lakes and rivers. Microbes, therefore, are also key members of the committee of stewards of planetary health and sustainability.

## Microbes provide a wide range of services to humans, animals and plants: microbiomes

Microbes cover the surfaces of all other organisms (and occupy internal and even intracellular niches, in some) and influence diverse physiological activities of their hosts, including nutrition, health–disease status and hence well‐being. The microbial flora of an organism is designated its microbiome. The microbiomes of food animals and crop plants regulate productivity, and thus global food production and quality. The human microbiome, which has been termed a human organ (Baquero and Nombela, 2012), in particular the intestinal microbiota, provides a host of metabolic and physiological services, and thereby has a pervasive positive influence on our well‐being, as we discover when their ordinarily benign networked activities become disrupted, e.g. by antibiotic treatment. Our intestinal flora helps digest our food and extract its nutritional content, and additionally provides us with essential nutrients we neither make ourselves nor take in via our diet. It also plays a pivotal role in the development of our immune system and profoundly influences its functioning, once developed (this is also the case in other animals and plants). On the negative side, a minority of microbes are pathogenic and able to cause disease. However, the earlier notion that infections result through acquisition of microbial pathogens is now moderated by the knowledge that many microbial pathogens are part of our normal, ordinarily benign flora, generally only causing problems when we or our microbiomes become perturbed. The microbiome is a principal regulator of the reproductive success of individual microbial populations and of functional groups of microbes on body surfaces, and hence of pathogen colonization events that precede development of disease. Microbial pathogen colonization/population expansion and subsequent interactions with target tissues and the immune system, which may or may not result in overt disease, are thus classical ecological processes, no more, no less. As a consequence, a systems understanding of the ecology of pathogens, particularly those with alternative lifestyles in the environment, is central to effective infection management and control. The current explosion in pathogen ecology research (see, e.g. recent Special Issue of Environmental Microbiology) reflects this. Recent findings suggest that intestinal microbiota may also profoundly influence brain function (e.g. see Sampson and Mazmanian, 2015) and are stimulating exceptionally exciting research at the interfaces of microbiology, neurobiology and psychiatry. This research promises to yield entirely new concepts in the perception of brain development and ageing, and the causes and management of mental ill health.

## Innovation and innovative solutions: harnessing microbes for an enormous spectrum of applications

**Figure 1:**
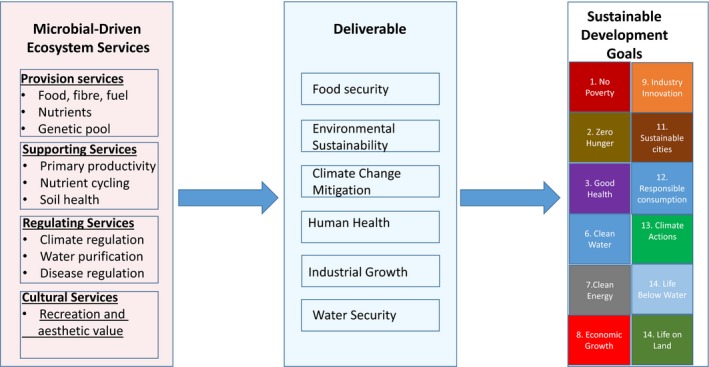
The Contributions of Microbial Biotechnology to Sustainable Development Goals. Microbial diversity and microbial technology are critical to achieve a majority of Sustainable Development Goals (SDGs), mainly due to the central role of microbes in the provision and regulation of ecosystem services. For example, microbial communities play a vital role in primary productivity, via nutrient cycling, and disease regulation, thereby impacting human, animal and plant health, and hence farm productivity and food security. Microbes and microbial technology are now increasingly employed in disease prevention and therapy, and to sustainably increase farm profitability, productivity and nutrient quality which directly contribute to SDGs 1, 2, 3. Similarly, microbes have critical roles in regulating climate via their contributions to both production and consumption of greenhouse gas (GHG) emissions and they create environments (e.g. soil structure) to support the growth of other organisms on land and water. A number of microbial technologies are used or in developmental pipeline for removal of GHGs and for purification of waste water for consumption. Microbes are a source of an exceptional range of chemicals and chemical catalysts, bioactive substances, biomaterials and of important forms of bioenergy, including hydrocarbons and electricity. They are key agents of pollutant removal and recycling. Microbes have been a source of industrial productivity throughout human history and currently are main drivers of the bioeconomy and industry, which are worth several trillion dollars, thereby contributing directly to SDGs 8 and 9.

Microbial activities and products have been employed in the service of mankind since the dawn of civilization (production of beer, cheese and fermented milk products, bread, wine, etc.). Although microbial technologies broadened in range over time, a quantum quantitative and qualitative increase was heralded in by the gene technology revolution in the 1970s. Over the current and coming decades, further quantum increases will be catalysed by a combination of an acceleration in accessing new microbial diversity, particularly though intensified exploration of our biosphere, (meta)genomics approaches, discovery and experimental creation of new types of metabolism and metabolic routes, new developments in analytical procedures, instrumentation and miniaturization (e.g. microfluidics), and increasing development and application of systems and synthetic biology. Innovation will not only be driven by progress in technology but also by commercial, medical and societal demands for diverse new and improved products and processes relating to food production and security, environmental protection and sustainable energy supplies. Exploration of microbiomes and their influence on human health, nutrition and disease is catalysing the evolution of new microbial application paradigms in disease prevention and therapy. And interdisciplinary research on plant and crop microbiomes is generating new knowledge with exciting potential for sustainable nutrient and disease management, and increased farm productivity and profitability (Fig. 1).

The vast range of unexplored microbial diversity and the pervasiveness of microbial activities influencing biosphere functioning and human endeavours and well‐being have recently precipitated a major expansion in environmental microbiology research. Despite this intensification of effort, more than 90% of microbial diversity still remains to be discovered. This new biodiversity represents a treasure trove of new and improved biotechnological developments and applications in the sectors of chemicals, pharmaceuticals, energy, mining, materials, agriculture, food and environmental protection. Moreover, the greater part of genomic information remains to be functionally decoded (*genomic dark matter*) and this also represents a wealth of potential applications, as exemplified by the successful activation of ‘silent’ determinants of secondary metabolites (e.g. see Schmidt‐Dannert, 2017; this issue). As the systematic exploration of microbial diversity and genetic space will reveal novel underlying catalytic reactions, metabolic activities and products, many of the applications resulting from these discoveries will be highly original and innovative. In particular, microbial life at the interface of the biosphere:geosphere, where it experiences extreme physico‐chemical conditions, is expected to exhibit unusual metabolic features, which will open up remarkable possibilities in terms of evolving catalytic activities (e.g. life processes in the Mariana trench take place at pressures of several hundred atmospheres with specific physico‐chemical start points that have not yet been explored). Moreover, natural microbial communities carry out cooperative metabolism in which pathways are highly branched and involve multiple, spatially organized, spatially interacting and spatially responding partners (e.g. see Pelz *et al*., 1999). Importantly, collaboration between microbes and distribution of reactions among different members of a community compartmentalize the chemistry and enables diverse metabolic activities to coexist that are considered to be functionally incompatible with one another.

## Microbial technologies contribute towards sustainable development in a broad range of key areas

Thus, the signature and almost unique characteristic of microbial technology is the exceptional diversity of applications it can address and the exceptional range of human activities and needs to which it is and can be applied (chemistry is another example). Precisely because sustainability goals have very diverse and complex components and requirements, microbial technology has the ability to contribute substantively on many levels in many arenas to global efforts to achieve sustainability (Fig. 1). Indeed, microbial technology could be viewed as a unifying element in our progress towards sustainability.

Nevertheless, individual areas of action in the diverse efforts towards sustainable development, and individual areas of microbial applications, tend to be viewed in isolation, rather than as interconnected and interdependent regions in a landscape continuum. As an explicit goal of this Journal is to proactively promote innovation in applied microbiology and the exploitation of discoveries in the service of mankind (e.g. see Special Issue Microbial Biotechnology‐2020, and Crystal Ball‐2017), its Editors and some friends of the Journal have compiled this Special Issue on Microbial Technology and Sustainability, in order to bring these fragmented parts together in a coherent landscape. We should, however, in all humility emphasize that the field of microbial biotechnology is exceptionally dynamic and that new applications are being discovered and developed on an almost weekly basis, so the landscape presented in this Special Issue can only be a snapshot illustration of the potential of the topic for sustainable development. Nevertheless, we believe that it provides a useful new perspective for future planning and policy and hope it will stimulate young people to engage in applied microbiological efforts to achieve sustainable development.

## Conflicts of interest

None declared.
